# Biomimetic Guided Bi_2_WO_6_/Bi_2_O_3_ Vertical Heterojunction with Controllable Microstructure for Efficient Photocatalysis

**DOI:** 10.3390/molecules28073123

**Published:** 2023-03-31

**Authors:** Yuanbo Sun, Ziang Jia, Ning Wan, Wei Feng

**Affiliations:** Key Lab of Groundwater Resources and Environment, Ministry of Education, Jilin University, Changchun 130021, China; ybsun21@mails.jlu.edu.cn (Y.S.);

**Keywords:** vertical heterojunction, control growth, bionics, Bi_2_WO_6_/Bi_2_O_3_, photocatalysis

## Abstract

To bridge the technical gap of heterojunction induction control in conventional semiconductor photocatalysts, a method of regulating the growth of heterojunctions utilizing biomimetic structures was designed to prepare a series of Bi_2_WO_6_/Bi_2_O_3_ vertical heterojunction nanocomposites for the disposal of environmentally hazardous tetracycline wastewater difficult to degrade by conventional microbial techniques. Porous Bi_2_O_3_ precursors with high-energy crystalline (110) dominant growth were produced using the sunflower straw bio-template technique (SSBT). Bi_2_WO_6_ with a (131) plane grew preferentially into 2.8 to 4 nm pieces on the (110) plane of Bi_2_O_3_, causing a significant density reduction between Bi_2_WO_6_ pieces and a dimensional decrease in the agglomerated Bi_2_WO_6_ spheres from 3 μm to 700 nm since Bi_2_WO_6_ grew on the structure of the biomimetic Bi_2_O_3_. The optimal 1:8 Bi_2_WO_6_/Bi_2_O_3_ coupling catalyst was obtained via adapting the ratio of the two semiconductors, and the coupling ratio of 1:8 minimized the adverse effects of the overgrowth of Bi_2_WO_6_ on degradation performance by securing the quantity of vertical heterojunctions. The material degradation reaction energy barrier and bandgap were significantly reduced by the presence of a large number of vertical heterojunction structures, resulting in a material with lower impedance and higher electron–hole separation efficiency; thus, the degradation efficiency of 1:8 Bi_2_WO_6_/Bi_2_O_3_ for tetracycline hydrochloride reached 99% within 60 min. In conclusion, this study not only successfully synthesized a novel photocatalyst with potential applications in water pollution remediation but also introduced a pioneering approach for semiconductor-driven synthesis.

## 1. Introduction

Tetracycline is one of the most widely used antibiotics in various countries due to its low price and broad-spectrum bactericidal properties, but factories discharge tetracycline wastewater that cannot be degraded by traditional microorganisms [1]. Hence, there is a need to develop low-cost and effective treatment technologies for treating highly concentrated tetracycline wastewater, as well as investigating their mechanisms of action [2,3]. Several inspiring solutions have been developed to address these serious issues [4,5]. One such technology, photocatalysis, is an advanced method for solving environmental and energy problems [6]. Scholars have developed numerous functional photocatalysts [7,8]. However, the photocatalysts can absorb light energy only in the UV range owing to their large bandgap [9,10]. In recent years, numerous novel materials that can obviate these weaknesses have been reported. For instance, Bi_2_O_3_ is well known for its polycrystalline characteristics [11,12,13,14], in which the stable α and γ phases plus the disintegratable β and δ phases are included [11]. Owing to its large dielectric constant, refractive index, good photoconductivity, and thermal properties, a wide range of applications exist for Bi_2_O_3_. It also possesses a bandgap between 2 eV and 3.96 eV [12], providing a wide spectral absorption range [13,14]. Nonetheless, the cyclic stability and catalytic efficiency of pure Bi_2_O_3_ are not satisfactory. Therefore, its material stability and catalytic efficiency require enhancement [15].

The use of composites comprising other semiconductor materials is an approach used to enhance the catalytic activity of Bi_2_O_3_ [16]. The solid–solid heterojunction interface can be formed at the contact surface of the two coupling semiconductors [17], which enables the semiconductor space to form a potential difference in close contact with the crystal boundary using the band overlap effect, accelerates the efficiency of charge separation within the system, and enhances the catalytic activity by expanding the spectral response range of the catalyst [18]. Despite the coupling of numerous different materials to Bi_2_O_3_ and the significant improvement in photocatalytic performance, some defects remain. Vertical heterogeneous structures with face-to-face inner surfaces are optimal for efficient charge transfer due to the wide and tight contact surface. Moreover, the two-dimensional (2D) blocks of the vertical heterojunction structure exhibit a specific confinement effect that increases the conversion rate of photon emission and narrows the diffusion of photogenerated charges [19]. Bi_2_WO_6_ tends to form flakes spontaneously under hydrothermal treatment and possesses the same Bi–O bond as Bi_2_O_3_; thus, Bi_2_WO_6_ can grow on the high-energy plane of Bi_2_O_3_ to form vertical heterojunction structures without the above defects.

The degradation effect of the Bi_2_O_3_/Bi_2_WO_6_ coupling catalyst was restricted via the controlling of crystal planes and heterojunctions in previous research [13]. Catalytic activity can also be improved by employing porous catalysts; however, an ordered pore structure merely increases the specific surface area of the material, which often results in unexpected consequences through utilizing naturally occurring biomimetic structures. Bio-templates enable hierarchical porous nanostructures to be conferred on photocatalytic materials and guide the growth of crystal surfaces [20,21]. Considering the abovementioned defects, we designed a hydrothermal synthesis method, coupling Bi_2_WO_6_ with SSBT-Bi_2_O_3_ and utilizing its biomimetic framework structure to prompt the growth of vertical heterojunction structures along a mass of high-energy planes [22]. The morphology and quantity of vertical heterojunction structures were visually characterized using TEM, XRD, and SEM. The growth points of the vertical heterojunction structures exposed to Bi_2_O_3_ (110) crystal planes and the distribution of vertical heterojunction structures in the modified sample were qualitatively analyzed. UV/Vis and PL spectroscopy were used to detect the bandwidth, electron–hole separation efficiency, and other properties to distinguish the structural performance improvement. Compared with the performance of other Bi_2_O_3_ materials in recent years, the material’s performance in this work was significantly improved [23,24,25,26,27].

## 2. Results and Discussion

### 2.1. Composition and Morphology

**Figure 1:**
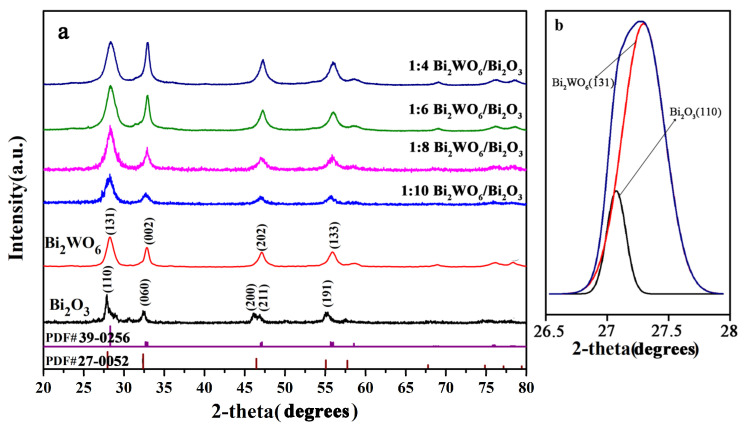
(**a**) XRD metal oxide patterns for Bi_2_O_3_, Bi_2_WO_6_, and Bi_2_WO_6_/Bi_2_O_3_ nanocomposite with different coupling rates. (**b**) Gaussian deconvolution peak fitting for Bi_2_O_3_ (110) and Bi_2_WO_6_ (131) of 1:8 sample.

Figure 1 shows the typical XRD patterns of Bi_2_WO_6_, Bi_2_O_3_, and Bi_2_WO_6_/Bi_2_O_3_ with different coupling ratios. According to the Standard cards (PDF#97-002-7150 and PDF#97-002-3584), all diffraction peaks fitted from both samples matched well with those of monoclinal β-Bi_2_O_3_ and orthomorphic tungsten-bismuth-type (attributed to layered perovskite) Bi_2_WO_6_. Planes of Bi_2_O_3_ and Bi_2_WO_6_ were maintained well in various coupled materials. Bi_2_O_3_ produced by SSBT exhibits a high-energy (110) plane [28,29]. No other characteristic diffraction peaks for impure compounds were observed, which confirmed the removal of the original compounds from the biological template. As shown in Figure 1b, the heterojunction recombination peak and the peaks of Bi_2_O_3_ (110) and Bi_2_WO_6_ (131) at 28.4° confirmed the formation of a 1:8 Bi_2_WO_6_/Bi_2_O_3_ heterostructure. In addition, the percentage of (110) planes was about 57.6% for SSBT-Bi_2_O_3_ crystals according to the XRD peak area calculation. The calculated content of (110) planes in the coupling materials was approximately equal to 9%. Only 1 in 10 coupling materials still accounted for 15.8% of the (110) plane, which was caused by the inadequate volume of Bi_2_WO_6_ in the coupling material.

**Figure 2:**
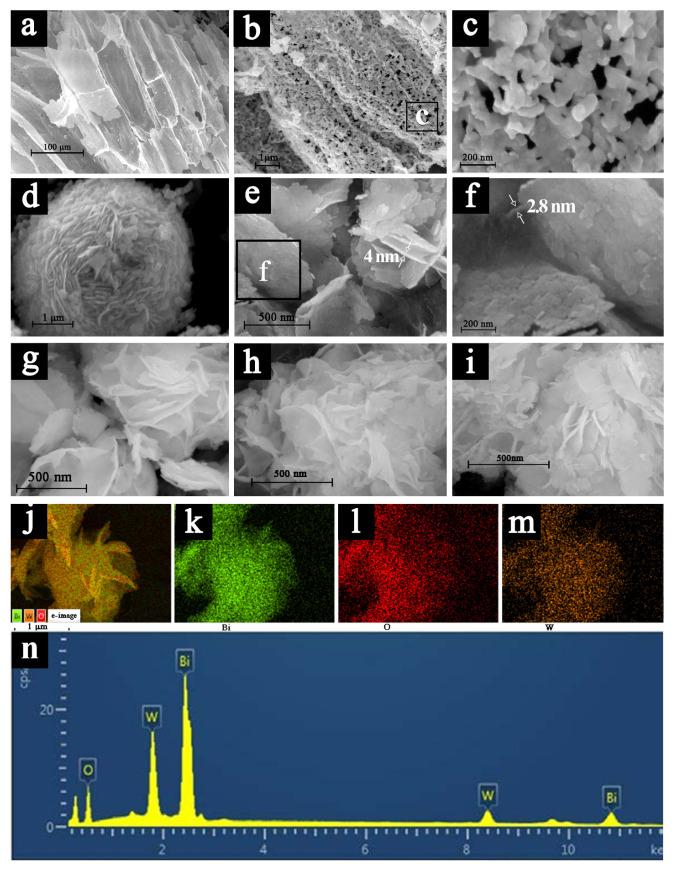
FESEM images of SSBT-Bi_2_O_3_ at different resolutions (**a**–**c**) and pure Bi_2_WO_6_ (**d**); SEM images with increasing resolution of 1:8 Bi_2_WO_6_/Bi_2_O_3_ (**e**,**f**), and 1:10 Bi_2_WO_6_/Bi_2_O_3_, 1:8 Bi_2_WO_6_/Bi_2_O_3_, and 1:6 Bi_2_WO_6_/Bi_2_O_3_ (**g**–**i**); mapping of 1:8 Bi_2_WO_6_/Bi_2_O_3_ (**j**–**m**) and the corresponding EDX (**n**). Note: (**c**,**f**) are enlargements of the boxed areas in (**b**,**e**).

Figure 2a–c show that SSBT-Bi_2_O_3_ successfully obtained the biomimetic structure of sunflower straw. The spherical floral structure in Figure 2d is pure Bi_2_WO_6_. Figure 2e–i show an unusual Bi_2_WO_6_ structure, which differed from the spherical agglomerates of pure Bi_2_WO_6_ due to the confinement growth of vertical heterojunction structures, where Bi_2_WO_6_ was tightly packed into 2.8–4 nm pieces and grown vertically on the (110) plane of Bi_2_O_3_. Therefore, the agglomeration was reduced, with a relative increase in active sites on the photocatalyst surface. We determined the surface areas of SSBT-Bi_2_O_3_, Bi_2_WO_6_, and 1:8 Bi_2_WO_6_/Bi_2_O_3_ using N_2_ adsorption as 45.17 m^2^/g, 32.42 m^2^/g, and 41.63 m^2^/g, respectively (Figure S2). All samples exhibited type IV adsorption isotherms with H2b hysteresis loops, which suggests a primarily mesoporous material, while SSBT-Bi_2_O_3_ featured a few macroporous structures due to the template. According to Figure 2g–i, materials with different coupling ratios differed in the distribution density of Bi_2_WO_6_ sheets at the macroscopic level. In addition, low-resolution graphs of SSBT-Bi_2_O_3_ with a full frame of the straw template and 1:4 Bi_2_WO_6_/Bi_2_O_3_ graphs are shown in Figure S1. Increasing the Bi_2_WO_6_ ratio after reaching the optimal active site density may reduce the performance of covering reactive sites.

**Figure 3:**
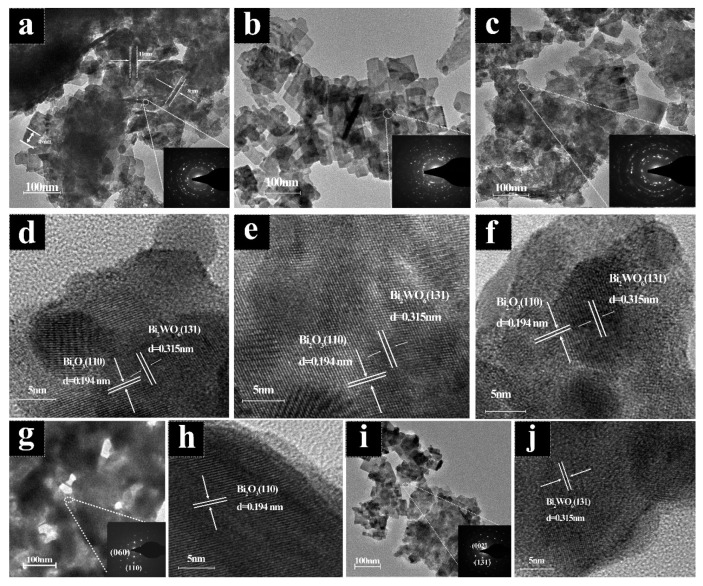
TEM images of 1:6 Bi_2_WO_6_/Bi_2_O_3_ (**a**), 1:8 Bi_2_WO_6_/Bi_2_O_3_ (**b**), and 1:10 Bi_2_WO_6_/Bi_2_O_3_ (**c**); HRTEM images of 1:6 Bi_2_WO_6_/Bi_2_O_3_ (**d**), 1:8 Bi_2_WO_6_/Bi_2_O_3_ (**e**), and 1:10 Bi_2_WO_6_/Bi_2_O_3_ (**f**); TEM and HRTEM images of SSBT Bi_2_O_3_ (**g**,**h**); TEM and HRTEM images of Bi_2_WO_6_ (**i**,**j**).

Figure 2j–m show the SEM element mapping of 1:8 Bi_2_WO_6_/Bi_2_O_3_ samples, indicating that the Bi_2_WO_6_/Bi_2_O_3_ composite mainly consisted of Bi, O, and W, which is consistent with the results of Figure 2n. The HRTEM images of Bi_2_O_3_ and Bi_2_WO_6_ in Figure 3h,j reveal distinct lattice fringes and lattice spacing, consistent with the XRD results above [30]. The size of Bi_2_WO_6_ grown on Bi_2_O_3_ differed under different coupling ratios. Bi_2_WO_6_ in Figure 3a had pronounced agglomeration, and the size and boundary were not noticeable, while the apparent growth of Bi_2_WO_6_ in Figure 3c was insufficient, with Bi_2_O_3_ accounting for the majority of sample. If coupling was excessive, agglomeration and stacking effects occurred, covering the heterojunction and reaction sites. Conversely, the growing size of Bi_2_WO_6_ was irregular, and the percentage of heterojunctions was insufficient. However, at a coupling ratio of 1:8, Bi_2_WO_6_ grew evenly on the Bi_2_O_3_ frame, with uniform and moderate sizes and no agglomeration effect (Figure 3b). From Figure 3d–f, we observed the Bi_2_O_3_ (110) high-index crystal plane and the bismuth tungstate (131) crystal plane, and the two grew interlaced to form a p–n heterojunction, coinciding with the XRD fitted peak crystal plane in Figure 1b. Combined with Figure 2e–i, it can be clearly seen that the slice layer Bi_2_WO_6_ grew on the hierarchical porous SSBT-Bi_2_O_3_ rather than being physically mixed; thus, the coupling of Bi_2_WO_6_ and Bi_2_O_3_ may form a heterostructure.

### 2.2. Spectroscopy and Electrochemical Characterization

**Figure 4:**
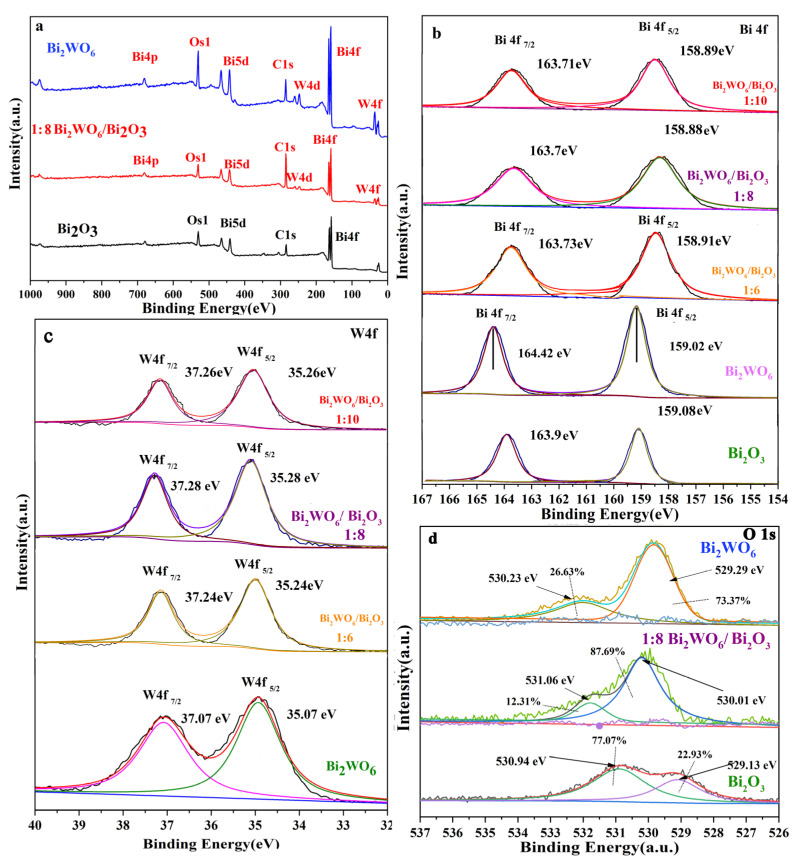
XPS spectra of Bi_2_WO_6_, 1:8 Bi_2_WO_6_/Bi_2_O_3_, and Bi_2_O_3_ (**a**); survey spectra for Bi 4*f* (**b**); W4f spectrum (**c**); O1 s spectrum (**d**).

The XPS spectrum in Figure 4a shows the elemental composition of each sample. Figure 4b shows that the binding energy for Bi 4*f* (Bi 4*f*_7/2_ and Bi 4*f*_5/2_) was 0.20 eV lower in the 1:8 Bi_2_WO_6_/Bi_2_O_3_ nanocomposite than in Bi_2_O_3_. Bi 4*f* spin–orbit split was similar to the data for Bi_2_O_3_ (4.82 eV), illustrating that Bi^3+^ displayed the same kinds of chemical states in the Bi_2_WO_6_/Bi_2_O_3_ nanocomposite and Bi_2_O_3_ [31]. However, the redshifts of 1:6 Bi_2_WO_6_/Bi_2_O_3_ and 1:10 Bi_2_WO_6_/Bi_2_O_3_ were 0.17 eV and 0.19 eV. As shown in Figure 4c, the W 4*f* XPS spectra for Bi_2_WO_6_ was divisible into two Gaussian deconvolution peaks at 37.07 eV and 35.07 eV, indicating that W was also present in the form of a positive hexavalent oxidation state in Bi_2_WO_6_ [32]. The binding energies of the W 4*f* Gaussian deconvolution peaks (W 4*f*_7/2_ and W 4*f*_5/2_) for the Bi_2_WO_6_/Bi_2_O_3_ nanocomposites were blue-shifted by approximately 0.21 eV (1:8), 0.17 eV (1:6), and 0.19 eV (1:10). According to the results in Figure 4b,c, the shift in the Gaussian deconvolution peaks was due to the heterojunction with the optimal coupling ratio absorbing and transferring electrons from the Bi–O bond to the W–O bond to the greatest extent. Therefore, the vertical heterojunction structure formed by the controlled growth allowed for extreme separation of photogenerated carriers in the 1:8 composite, as well as dramatically increasing the proportion of vacant O in the coupling material to 87.69%, which is much higher than that of any single material [30]. This entailed the combination of oxygen and photogenerated electronics to form a large amount of •O^2−^. Subsequently, •O^2−^ and H_2_O combined to generate •OH, which facilitated photocatalytic excitation and promoted photocatalytic performance.

**Figure 5:**
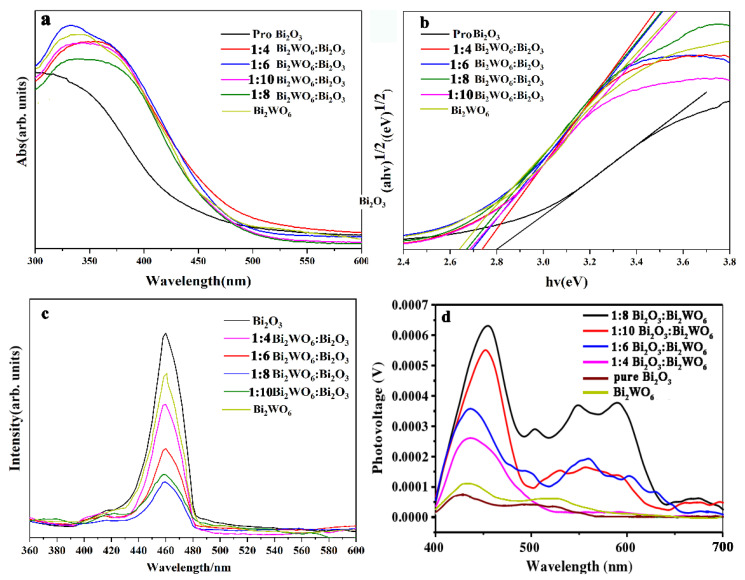
UV/Vis DRS (**a**) and plots of (αhv)^1/2^ versus hv (**b**) of samples; PL spectra (**c**) and SPS surface photovoltage spectra (**d**) of samples.

In addition, the absorption edge of the Bi_2_WO_6_/Bi_2_O_3_ sample had a different degree of redshift in the absorption level compared to the Bi_2_O_3_ sample, especially for the 1:8 coupling ratio. The heterojunction structure of the best-matched material should have a small bandgap to further estimate the specific bandgap values of the prepared samples according to the reported methods (Kubelka–Munk equation (ahv)^2^ = A^2^(hv − Eg)) (the letter definition in Kubelka–Munk equation: αhν: absorption coefficient; A: proportionality constant; hν: photon energy, where h is Planck’s constant and ν is the light frequency; Eg: bandgap energy of the material) Butler for crystalline semiconductor studies (Table S1) and the VB calculated via XPS (Figure S3). Since Bi_2_WO_6_ and Bi_2_O_3_ are typical indirect bandgap semiconductors [33,34], we obtained optical bandgap values for Bi_2_WO_6_ (2.66 eV) [35], Bi_2_O_3_ (2.80 eV) [36], 1:4 Bi_2_WO_6_/Bi_2_O_3_ (2.74 eV), 1:6 Bi_2_WO_6_/Bi_2_O_3_ (2.69 eV), 1:8 Bi_2_WO_6_/Bi_2_O_3_ (2.67 eV), and 1:10 Bi_2_WO_6_/Bi_2_O_3_ (2.68 eV) using the plots of (αhv)^1/2^ versus hv (Figure 5a,b).

**Figure 6:**
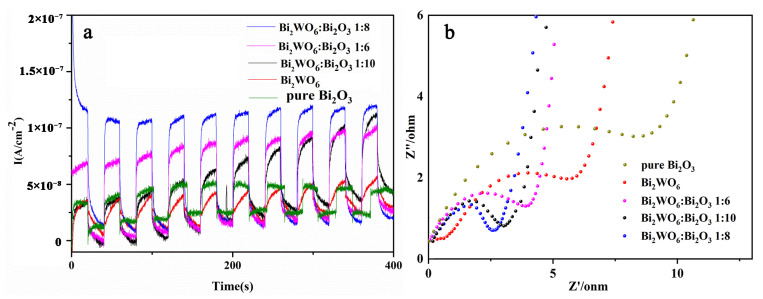
Photocurrent response (**a**) and EIS spectra (**b**) of Bi_2_O_3_ and Bi_2_WO_6_/Bi_2_O_3_ at different ratios.

The hybridization of the 4*f* state of W and Bi_2_O_3_ widened the valence band, resulting in a narrower bandgap. However, due to the higher bandwidth of Bi_2_WO_6_, the excessive coupling may have caused the composite bandwidth to increase [37]. The lowest energy bandgap could be obtained by controlling the heterojunction structure with a coupling ratio of 1:8. To further verify the above conclusion, we tested the room-temperature PL spectra (Figure 5c) and surface photovoltage spectroscopy (SPS Figure 5d) of the as-prepared samples. As shown in Figure 5c, Bi_2_O_3_ and Bi_2_WO_6_/Bi_2_O_3_ exhibited a clear diffraction peak at 460 nm, which matches the UV/Vis results. The PL emission intensity of Bi_2_WO_6_/Bi_2_O_3_ was significantly lower than that of Bi_2_O_3_, especially 1:8 Bi_2_WO_6_/Bi_2_O_3_. In addition, Figure 6b shows that the EIS radius of Bi_2_O_3_ was the largest, and the EIS radius of 1:8 Bi_2_WO_6_/Bi_2_O_3_ was significantly smaller than the others, indicating that the 1:8 Bi_2_WO_6_/Bi_2_O_3_-controlled heterojunction structure could minimize the recombination efficiency of photogenerated electron holes. In addition, according to Figure 5d and Figure 6a, the relative strength of the SPS signal response and transient photocurrent (Figure 6a) strength of the prepared samples was in the order pure Bi_2_O_3_ < Bi_2_WO_6_ < 1:4 Bi_2_WO_6_/Bi_2_O_3_ < 1:6 Bi_2_WO_6_/Bi_2_O_3_ < 1:10 Bi_2_WO_6_/Bi_2_O_3_ < 1:8 Bi_2_WO_6_/Bi_2_O_3_. The above data show that the best ratio (1:8) of heterojunctions can utilize visible light to a larger extent than pure Bi_2_O_3_. The heterojunction structure with the optimal ratio could maximize the separation of photogenerated electron–hole pairs. This agrees with the previous PL characterization analysis.

### 2.3. Photocatalytic Degradation of Tetracycline

**Figure 7:**
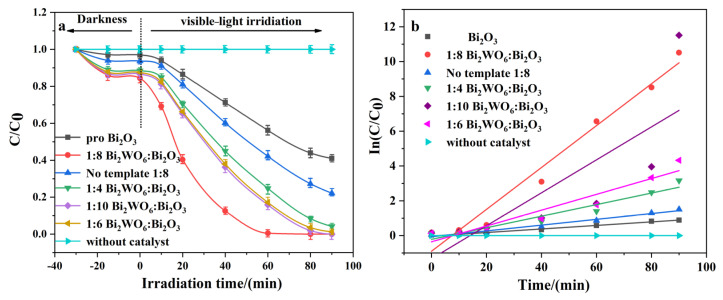
Photocatalytic activity to 20 mg/L TCH by 0.4 g catalyst (**a**); primary reaction kinetic curves for degradation processes (**b**).

In order to carry out a rational analysis of the material, degradation performance was first evaluated, as shown in Figure 7a; the TOC is shown in Figure S4. Photocatalytic degradation of TCH under visible light (pH = 6) reached the adsorption equilibrium in 30 min without light, and TCH was not significantly degraded. The degradation rate of pure SSBT−Bi_2_O_3_ was 60% at an initial TCH concentration of 20 mg/L and 90 min of visible-light irradiation. The degradation properties of the composites were greatly enhanced by introducing Bi_2_WO_6_, especially 1:8 Bi_2_WO_6_/Bi_2_O_3_. This may have been due to the heterojunction formed by the B–O bond of Bi_2_WO_6_ and Bi_2_O_3_, which helped to separate the photogenerated carriers and narrowed the bandgap, as proven by the subsequent spectral characterization. In addition, the Bi_2_WO_6_/Bi_2_O_3_ nanocomposites with different coupling ratios of 1:4, 1:6, and 1:10 achieved TCH degradation efficiencies of 96%, 97%, and 99% after 90 min of light, respectively. Nevertheless, 1:8 Bi_2_WO_6_/Bi_2_O_3_ only took 60 min to reach a degradation rate close to 100%, performing multiples of the similar Bi_2_O_3_ composite catalysts (Figure S8).

**Figure 8:**
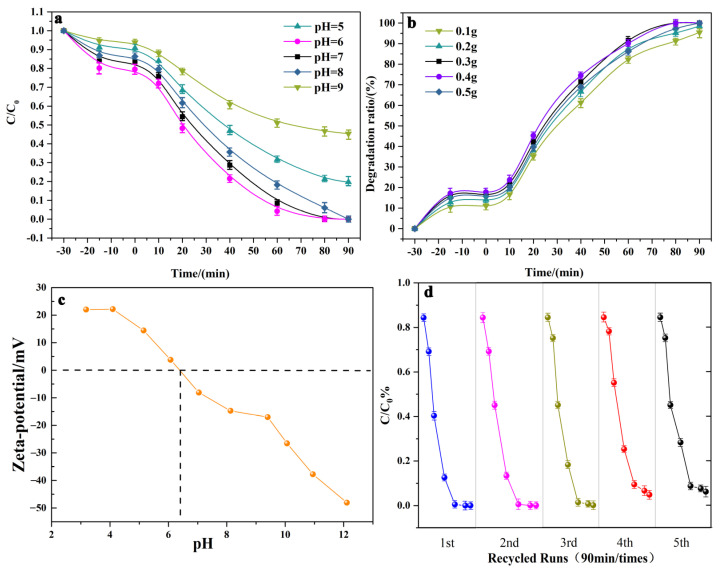
Degradation effect of 1:8 Bi_2_WO_6_/Bi_2_O_3_ under different pH values and catalyst dosage (**a**,**b**); zeta potential and cycling stability of 1:8 Bi_2_WO_6_/Bi_2_O_3_ (**c**,**d**).

The photodegradation process followed first-order kinetics, with the constant rate k being the slope of the fitted line (reaction rate), as shown in Figure 7b [30]. Specifically, 1:8 Bi_2_WO_6_/Bi_2_O_3_ had the largest photodegradation rate constant, 90% higher than pure Bi_2_O_3_. Therefore, the best-performing 1:8 Bi_2_WO_6_/Bi_2_O_3_ was chosen as the core characterization material for the subsequent experiments. As shown in Figure 8d, after five consecutive cycles of photocatalytic degradation of tetracycline, the degradation rate continued to be at a high level until the fifth cycle, whereby there was a degradation rate of 96% and no significant change in nanostructure (Figure S5). This shows that the catalyst can be efficiently recycled. The above characterization experiments referenced the optimal conditions in the performance test. In addition, tests of the photocatalytic effect under different pH values and catalyst dosing were performed (Table S3), showing that the optimum pH was 6 and the catalyst dosing was 0.4. The zeta potential of the composite material under different pH conditions was measured as shown in Figure 8c, and the isoelectric point of the material was 6.4. The catalyst surface was positively charged when the pH of the solution was below the isoelectric point of the material; otherwise, it was negatively charged. The dissociation constants of TCH are pKa1 = 3.3, pKa2 = 7.7, and pKa3 = 9.7. Tetracycline in solution exists as a zwitterion when 3.3 < pH < 7.7 [38]. Therefore, when the catalyst and tetracycline have different charges, they attract each other, which can improve the removal efficiency in respect of pollutants. The removal rate for tetracycline reached the maximum at pH = 6. However, the ionization degree of the phenolic hydroxyl group of TCH increased due to the high OH^−^ concentration, which weakened the formation of coordination compounds containing Bi and, thus, reduced the enrichment of TCH in the material. However, the increase in H^+^ concentration inhibited the formation of •OH, and the protonation rate of amino groups was higher, which increased the co-ion repulsion effect between TCH near the material and reduced the adsorption effect, which are not conducive to the photocatalytic reaction.

### 2.4. Mechanisms

**Figure 9:**
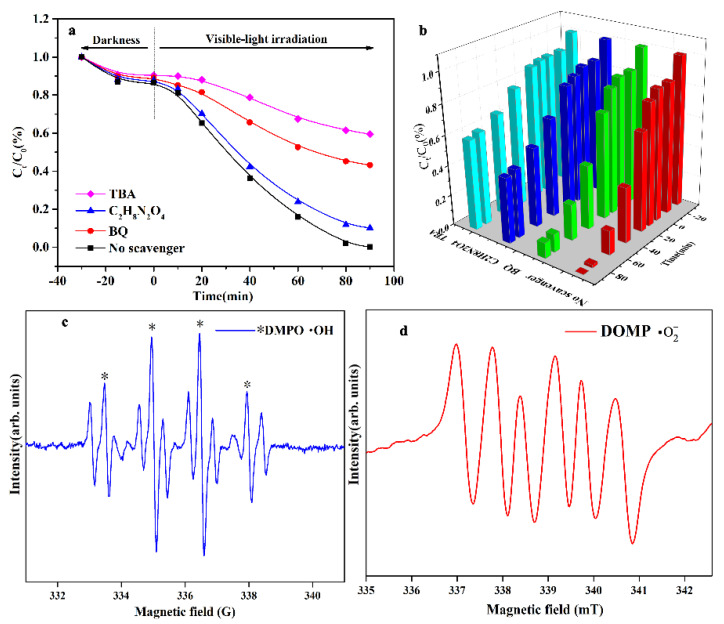
The 1:8 photocatalytic material system: photocatalytic efficiency of TCH with different scavengers under visible-light irradiation (**a**,**b**); ESR spectra of DMPO-•OH and -•O_2_^−^ under aqueous and methanol phase (**c**,**d**).

To identify the reactive radicals that play a significant role in the degradation of this material system and to further explain the reaction mechanism, degradation pathway analysis and free-radical scavenging experiments were carried out. The results in Figure 9a,b show that the addition of all three free-radical scavengers had some effect on the degradation efficiency. After adding TBA, •OH radicals were suppressed, and the degradation efficiency decreased the most, followed by BQ, with C_2_H_8_N_2_O_4_ at the bottom; the order of influence of the three capture agents was TBA (•OH) > BQ •(O_2_^−^) > C_2_H_8_N_2_O_4_ (h^+^), indicating that all three free radicals contributed to the degradation of TCH. In comparison, •OH was the active substance playing the most prominent role, followed by •O_2_^−^, with h^+^ playing the least prominent role. To confirm the accuracy of the above conclusions and to directly prove the presence of free radicals, we performed an EPR (electron paramagnetic resonance) free-radical assay, as shown in Figure 9c,d. A typical fourfold OH peak (Asterisk *) with an intensity ratio of 1:2:2:1 was detected in the ESR spectrum, indicating that •OH was generated during the reaction [39]. Furthermore, the sixfold peak of •O_2_^−^ evidenced the presence of •O_2_^−^ [40].

**Figure 10:**
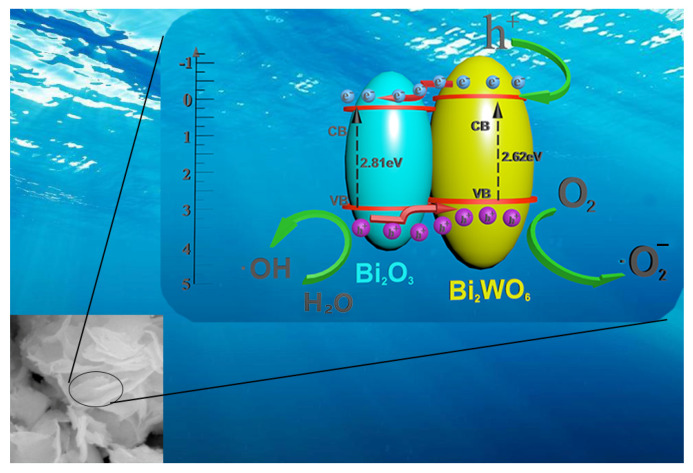
The 3D schematic diagram of the heterojunction structure and photocatalytic mechanism of the Bi_2_WO_6_@Bi_2_O_3_ heterojunction composite catalyst.

Figure S6 shows the mass spectrum of the water sample detection of TCH in the presence of 1:8 Bi_2_WO_6_@Bi_2_O_3_ catalyst at different degradation times. Figure S6a shows that the chromatogram of the 0 min reaction was the characteristic peak of TCH with a retention time of 5.01 min. The intensity of this peak gradually decreased with the increase in photocatalytic reaction time. A well-defined peak shape was still found after 15 min of reaction (Figure S6b). After 45 min (Figure S6c) and 60 min (Figure S6d), the intensity of the peaks decreased significantly, accompanied by a large number of faint pseudo-peaks, indicating the gradual degradation of TCH to small molecular particles. According to the charge-to-mass ratio data for TCH at each reaction time, the possible molecular structures of some products in the degradation process were analyzed, and the degradation pathways were drawn (Table S2 and Figure S7). Considering the previous characterization, we deduced the following mechanism: when two semiconductors with different work functions come into contact, electrons are transferred from the material with the higher Fermi level to the material with the lower Fermi level until both have the same Fermi level [41]. As shown in Figure 10, the band alignment of Bi_2_O_3_ and Bi_2_WO_6_ exhibited type II alignment. Before contact, the conduction and valence bands of Bi_2_O_3_ were higher than those of Bi_2_WO_6_. The Fermi energy level of the p-type semiconductor Bi_2_O_3_ was lower than that of the n-type semiconductor Bi_2_WO_6_, facilitating the separation of photogenerated electron and hole pairs [42]. When the two semiconductors were in close contact to form a p–n heterojunction, the electrons first flowed from Bi_2_WO_6_ with a high Fermi level to Bi_2_O_3_ with a lower Fermi level. The CB and VB of Bi_2_WO_6_ moved downward, while the CB and VB of Bi_2_O_3_ moved upward until the Fermi levels of the two reached a new equilibrium state. The absorption edge of the optimal vertical heterojunction had the largest blueshift, which may have been due to the heterojunction structure under the best control effect, which enabled the W–O bond to absorb and transfer Bi–O bond electrons to the greatest extent. Hence, the bandgap value of 1:8 sample was also the smallest. Therefore, an internal electric field was formed between the p–n heterojunction interfaces in the optimal regulation ratio, as shown in Figure 10. Bi_2_WO_6_/Bi_2_O_3_ heterojunction catalysts were excited under visible light; photogenerated electrons and holes were generated; and the electrons transitioned. Affected by the internal electric field, photogenerated electrons migrated from the CB of Bi_2_O_3_ to the CB of Bi_2_WO_6_. The photogenerated holes on the Bi_2_WO_6_ VB were transferred to the Bi_2_O_3_ VB, promoting the separation of photogenerated electron–hole pairs and suppressing their recombination [43]. The optimal vertical heterojunction had a narrower bandgap, facilitating the separation of electrons and holes. The above results are also consistent with the PL and EIS tests. Subsequently, the electrons collected on the conduction band of Bi_2_WO_6_ were captured by the oxygen adsorbed on the heterojunction surface. The reaction generated superoxide radicals (•O_2_^−^), which were protonated to generate •OH [44]. The holes in the valence band and H_2_O and OH^−^ also generated •OH. Eventually, •OH, •O_2_^−^, and holes became active materials with oxidizing solid abilities, which can degrade pollutants. When the optimal coupling ratio of 1:8 was reached, the (131) crystal plane of Bi_2_WO_6_ and the (110) crystal plane of Bi_2_O_3_ achieved the best combination ratio. As the coupling rate continued to increase, agglomeration and stacking effects covered the heterojunction and reaction sites. The bandwidth also expanded again due to the broader bandwidth of Bi_2_WO_6_. If the coupling ratio was too small, the growth size of Bi_2_WO_6_ was irregular and small, and the number of heterojunctions was insufficient. Only when the two semiconductors were in the best ratio (1:8) was the energy band in the heterojunction in the most suitable bending range (band bending). The photogenerated carriers were highly separated, facilitating the electronic transition. The faster electron separation efficiency led to the enhancement of heterojunction performance (photocatalytic performance). This was manifested by the low photogenerated electron–hole recombination rate, as also confirmed by the high conductivity shown in the EIS results.

## 3. Conclusions

In this study, we enabled semiconductor catalysts to obtain hierarchical porous biomimetic structures, and we directed the dominant growth of high-energy planes (Bi_2_O_3_(110)) by releasing agricultural waste. In a novel approach, the coupling ratio and the dominant plane were utilized to control the confined growth of the coupled material such that the vertical heterojunction structure was uniformly distributed in the material. The best combination ratio of the (131) crystal plane of Bi_2_WO_6_ and (110) crystal plane of Bi_2_O_3_ was achieved when the coupling ratio was 1:8. A further increase or decrease in the coupling rate affected the number of photocatalytic active sites, thus leading to a decrease in catalytic efficiency. The optimally proportioned vertical heterojunction material featured a tiny bandgap of 2.67, and its photocatalytic degradation of TCH reached 99% within 60 min. Compared with the performance of other Bi_2_O_3_ materials in recent years, the performance of this material was improved severalfold. A new method for regulating the growth of vertical heterojunction structures was successfully developed in this study, which can provide new ideas for synthesizing photocatalytic materials for water treatment. The biotemplate selected in this work shows a low specific surface area, and further work will be carried out to select natural photonic crystal biotemplates rich in micropores to fabricate catalysts with better performance.

## 4. Materials and Methods

### 4.1. Chemicals

Bi(NO_3_)_3_·5H_2_O, Na_2_WO_4_·2H_2_O absolute ethanol (CH_3_CH_2_OH), nitric acid (HNO_3_), and ammonia (NH_3_) were purchased from Sinopharm Chemical Regent Co., Ltd. (Shanghai, China). The above reagents can be used directly (all are analytical grade). Tetracycline hydrochloride (CH_22_H_24_N_2_O_8_·HCl) was of biotechnology grade and purchased from Shanghai McLean Biochemical Technology Co., Ltd. (Shanghai, China). Sunflower stalks are sourced from local suburban farms.

### 4.2. Material Synthesis

#### 4.2.1. Pretreatment of Sunflower Stalk

First, the stiffer outer skin of the sunflower stalk was peeled off, leaving a tissue with a porous structure inside. It was then cut into pieces with a thickness of 1 to 2 mm and dried for use. Due to the presence of grease, filling tissue, wax, and other substances, a large amount of lignin, cellulose, and hemicellulose were cross-linked and mixed in the straw, resulting in the clogging of the stalk pores. Therefore, the stalk needs to be pretreated to improve its impregnation performance. Currently, more commonly used pretreatment methods were the lye immersion and ammoniation [45]. The stalk pieces were placed in a Soxhlet extractor, extracted for about 5 h, then repeatedly washed with distilled water to a pH of about 7, dried in an oven at 90 °C for 12 h, and set aside.

#### 4.2.2. Preparation of Bi_2_O_3_ by Biological Template

Prepare 100mL solution of bismuth 20% nitrate and weigh 9.7 g Bi(NO_3_)_3_ into 0.1 mol/L nitrate. The pretreated sunflower stalk tissue is immersed in the configured Bi(NO_3_)_3_ solution. Let the diced completely immersed in the solution and seal the beaker. Then, pour off the solution after 24 h and rinse repeatedly with anhydrous ethanol about 3 times to remove the surface-adhered Bi(NO_3_)_3_ solution. Each batch of sunflower stalks is immersed twice to saturate; then, the impregnated sunflower stalk are divided into a tube furnace; the heating rate is set at 2 °C/min. After heat reaches a temperature of 300 °C, it stays there for 1 h, then passing the heating rate of 1 °C/min to 450 °C and then staying there for 5 h to complete removal of the sunflower stalk template to obtain pure Bi_2_O_3_.

#### 4.2.3. Preparation of Bi_2_WO_6_/Bi_2_O_3_

Molar ratios of 1:2:4, 1:2:6, 1:2:8, and 1:2:10 were weighed Na_2_WO_4_·2H_2_O, Bi(NO_3_)_3_, and SSBT-Bi_2_O_3_, respectively. After the Na_2_WO_4_·2H_2_O solution is dissolved in 40mL of distilled water after Na_2_WO_4_·2H_2_O is completely dissolved, Bi_2_O_3_ is added to the Na_2_WO_4_·2H_2_O solution, and the Bi(NO_3_)_3_ solution is dissolved in 20 mL of 0.1 mol/L nitric acid solution and sonicated for 30 min, and Bi_2_O_3_ is completely dispersed in the solution. In the mixture, the dissolved Na_2_WO_4_·2H_2_O solution was added to the mixture and rapidly stirred, and the ultrasonication was continued for 30 min to disperse the mixture uniformly. After ultrasonication, the mixed solution was poured into a 100 mL reaction vessel and placed in an oven at 160 °C for 24 h. After the temperature of the oven was lowered to room temperature, the reaction kettle was taken out, and the mixture was centrifuged at 8000 r/min for 30 min to remove the supernatant, and the precipitate was repeatedly washed three times with distilled water and absolute ethanol to remove surface-adsorbed impurities and ions. Finally, the solid was placed in a 60 °C oven to obtain Bi_2_WO_6_/Bi_2_O_3_ composites with different doping ratios. The molar doping ratios of Bi_2_WO_6_/Bi_2_O_3_ were 1:4, 1:6, 1:8, and 1:10. In addition, a hydrothermal reaction without bismuth oxide for comparison is prepared. This sample is pure Bi_2_WO_6_.

### 4.3. Characterization

We used X-ray diffractometry to characterize the crystal structure of the samples with the Cu-Kβ irradiation source at a scanning rate (2θ) of 10° min^−1^ from 10° to 90°. The morphology was measured using a field emission scanning electron microscope (SEM, XL-30, FEI Inc., Valley City, ND, USA) and a JEM-200CX transmission electron microscope (TEM, JEM-200CX, Electronics Co. Kyoto, Japan). A U-4100 UV-visible spectrophotometer (U-4100, Shimadzu Co., Kyoto, Japan) was used to characterize the light absorption performance of the sample in the UV–visible region. Based on BaSO_4_, the scanning wavelength range was 200–800 nm. A FLS-980 fluorescence spectrometer (PL, FLS-980, Edinburgh Instruments Co., Livingston, UK) was used to examine the photoluminescence properties of the samples. The excitation source was a xenon lamp with an excitation wavelength of 300 nm and a slit width of 1 nm. X-ray photoelectron spectroscopy (XPS) was performed using a Thermo ESCALAB 250XI spectrometer with an Al Kα source. High-performance liquid chromatography (HPLC) (ExionLC, Shimadzu) was combined with triple quadrupole tandem mass spectrometry (MS/MS) (AB API4500Q, SCIEX, Framingham, MA, USA) for different degradation times of organic pollutants under the reaction. The samples were analyzed by HPLC-MS/MS. The operating conditions of the mass spectrometer were as follows: liquid chromatography was carried out on an Agilent SBC18 (2.1 × 100 mm, 3.5 μm) column. The column temperature was 30 °C, and the flow rate was 0.25 mL/min. Mobile phase A was a mixture of 5% acetonitrile and 95% formic acid (1 ‰, *v*/*v*), and mobile phase B was a mixture of 80% acetonitrile and 20% formic acid. (1 ‰, *v*/*v*). Mobile phase A was used at 0, 1, 12, and 15 min, and mobile phase B was used at 7 and 10 min. The injection volume was 2 μL. Mass spectrometry was performed in positive ionization (ESI) mode with an ionization voltage of 5.5 kV, desolvated gas at 0.75 MPa, atomized gas at 0.625 MPa, auxiliary gas at 0.625 MPa, collision gas at 0.15 MPa, and ion source temperature at 550.0 °C. The full scan mode range was 50–800 Da (*m*/*z*). The surface photovoltage spectrum (SPS) was calibrated using a silicon detector (DSI200, Zolix, Beijing, China) in the test system. The detection scan ran from long waves to short waves, and when the wavelength was greater than 600 nm, the filter (>420 nm) was used to remove the frequency-doubled light. Qualitative detection of •OH and •O_2_^−^ in the reaction process used electron spin resonance (ESR) (Bruker A200 Munich, Germany). Detection conditions: central field strength of 3360 G; scan width of 100 G; microwave frequency of 9.75 GHz; the power of 6.33 mW.

### 4.4. Catalytic Performance Testing of Materials

The photodegradation properties of the prepared material were demonstrated by degrading TCH in visible light. A 150 W xenon lamp (400~800 nm) was used as the light source, and 20 mg/L tetracycline was used as the target pollutant. The photocatalytic reaction process was as follows: weigh a certain amount of catalysts (SSBT- Bi_2_O_3_, Template-free Bi_2_WO_6_/Bi_2_O_3_, different ratio of Bi_2_WO_6_/Bi_2_O_3_) into a 250 mL beaker. Then, add 250 mL of a 20 mg/L tetracycline solution and use H_2_SO_4_ and NaOH solution to adjust the pH value of the solution. Start the magnetic stirrer (rotation speed 700 r·min^−1^). Turn on the xenon light source after 30 min adsorption in the dark state and start timing. Sampling was performed at a certain interval (15 min, 30 min, 10 min, 20 min, 40 min, 60 min, 80 min, 90 min) and filtering with a 0.45 μm filter to measure the absorbance of the sample at a wavelength of 357 nm with a UV–visible spectrophotometer. The initial concentration of tetra-TCH and the change in concentration after degradation were recorded according to the variation of absorbance, and their ratio was the degradation rate. In addition, several repeated experiments were conducted to test and record the effects of different catalyst dosages, temperatures, pH values, and repeated use times on the catalytic degradation performance. The experimental data used in the drawing were the average values of 5 repeated experiments after excluding error values. Samples were recovered by filtration at the end of each experiment, washed with ethanol and water, dried, and used for the next repeat experiment. Reactive radical detection is performed by the following steps: excess (250 mmol/L) tert-butanol, p-benzoquinone, and ammonium oxalate were used as inhibitors of •OH, •O_2_^−^, and holes (h^+^), respectively [46,47]. The active species can be detected by Electron spin resonance (ESR) using 5,5-dimethyl-1-pyrroline N-oxide (DMPO) as a free radical trapping agent. Based on the degradation rate of tetracycline, the existence of the above three kinds of free radicals and the free radicals which play a major role is evaluated.

## Data Availability

The datasets generated and/or analyzed during the current study are available from the corresponding author upon reasonable request. For non-copyright or non-privacy restricted data, please contact the corresponding author for access.

## Supplementary Materials

The following supporting information can be downloaded at https://www.mdpi.com/article/10.3390/molecules28073123/s1: Figure S1. FESEM images of complete structure of SSBT- Bi_2_O_3_ at low magnification (a) and 1:4 Bi_2_WO_6_/Bi2O_3_ (b); Figure S2. N2 adsorption-desorption isotherms and pore distribution (In-Picture Diagram) of SSBT- Bi_2_O_3_ (a), Bi_2_WO_6_ (b) and 1:8 Bi_2_WO_6_/Bi2O_3_ (c); Figure S3. VB XPS spectra of different samples; Figure S4. Comparison chart of TOC removal rate between different materials; Figure S5. SEM (a) and TEM (b) of 1:8 Bi_2_WO_6_/Bi2O_3_ after 5 cycles of degradation experiments; Figure S6. HPLC-MS/MS chromatograms of Tetracycline Hydrochloride degradation products with 1:8 Bi_2_WO_6_/Bi2O_3_ catalyst: (a) 0 min; (b) 15 min; (c) 45 min; (d) 60 min; Figure S7. Proposed degradation pathway of TCH with the Bi_2_WO_6_/Bi_2_O_3_ catalyst; Figure S8. Degradation time of tetracycline by the 1:8 Bi_2_WO_6_/Bi2O_3_ of this work versus the same type of Bi_2_O_3_ composite catalyst in other works; Table S1. X, Eg, ECB and EVB of all samples. Table S2. Proposed degradation pathway of TCH with Bi_2_WO_6_/Bi2O_3_ catalyst; Table S3. Changes in the pH of the solution during the degradation process of 1:8 Bi_2_WO_6_/Bi2O_3_ catalyst.

## Sample Availability

Samples of the compounds are available from the authors.
