# Supplementary material for: Biomimetic Guided Bi2WO6/Bi2O3 Vertical Heterojunction with Controllable Microstructure for Efficient Photocatalysis
Source: Molecules. 2023 Mar 31;28(7):3123. doi: 10.3390/molecules28073123 (PMC10096349; doi:10.3390/molecules28073123)
Supplement: Supplementary file 1 [file molecules-28-03123-s001.zip › molecules-2271723-supplementary.pdf]

Calculation of band positions

The band positions of Bi_2O_3 and Bi_2WO_6/Bi_2O_3 can be calculated by the following empirical formulae:

$$E_{CB} = X - E_c - 1/2E_g, E_{VB} = E_{CB} + E_g$$

where X is the absolute electronegativity of the atom semiconductor, expressed as the geometric mean of the absolute electronegativity of the constituent atoms, which is defined as the arithmetic mean of the atomic electron affinity and the first ionization energy; E_c is the energy of free electrons of the hydrogenscale (~ 4.5 eV); E_g is the band gap of the semiconductor; E_{CB} is the conduction band potential and E_{VB} is the valence band potential.

Semiconductor	X (eV)	E_g (eV)	E_{CB} (eV)	E_{VB} (eV)
Bi_2O_3	6.20	2.80	0.295	3.095
1:4 Bi_2WO_6/ Bi_2O_3	6.20	2. 737	0.3315	3.0685
1:6 Bi2WO_6/ Bi_2O_3	6.20	2.695	0.3525	3.0475
1:8 Bi_2WO_6/ Bi_2O_3	6.20	2.678	0.361	3.039
1:10Bi_2WO_6/Bi_2O_3	6.20	2.684	0.358	3.042
Bi_2WO_6	6.20	2.661	0.442	3.103

Table-S1 X, E_g, E_{CB} and E_{VB} of all samples

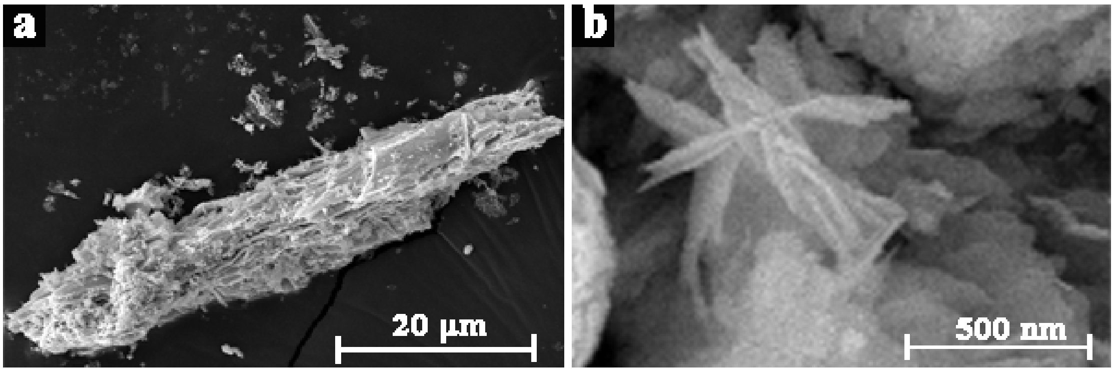

Figure S1 FESEM images of complete structure of SSBT- Bi_2O_3 at low magnification （a） and 1:4 Bi_2WO_6/Bi_2O_3（b）

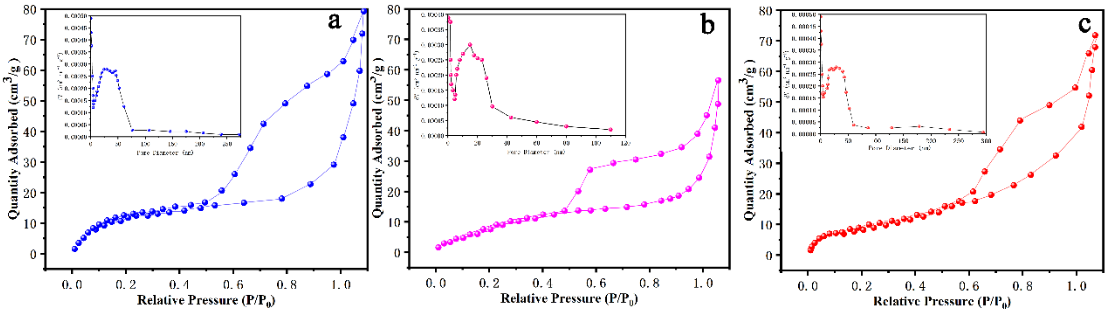

Figure S2 N_2 adsorption-desorption isotherms and pore distribution (In-Picture Diagram) of SSBT- Bi_2O_3(a), Bi_2WO_6(b) and 1:8 Bi_2WO_6/Bi_2O_3 (c)

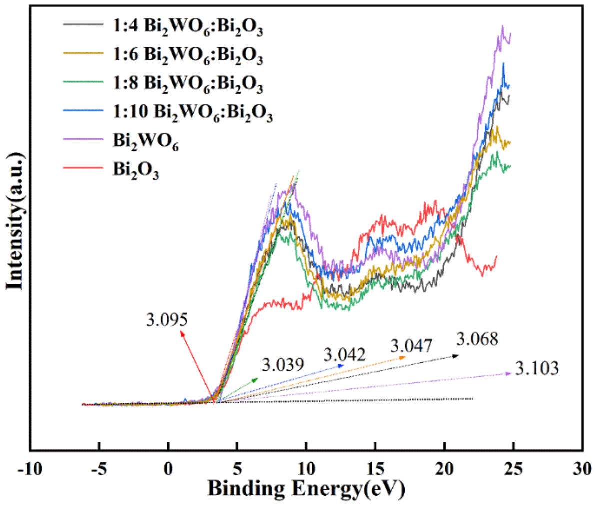

Figure S3 VB XPS spectra of different samples

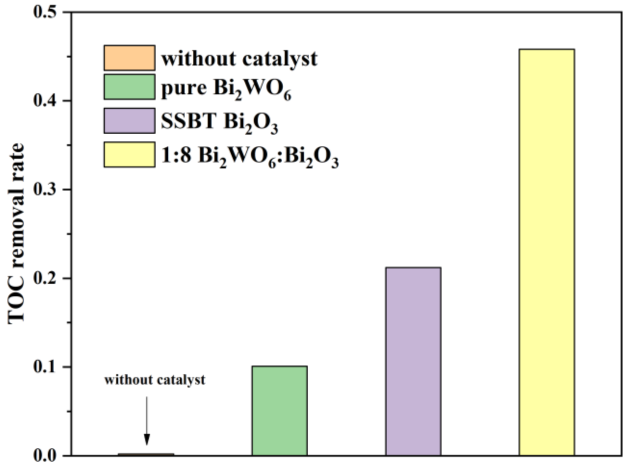

Figure S4 Comparison chart of TOC removal rate between different materials

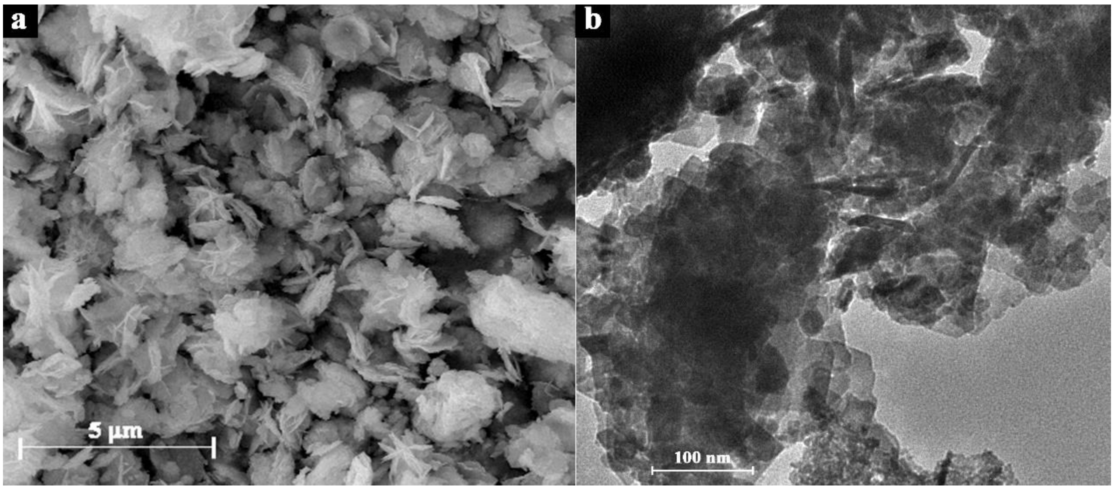

Figure S5 SEM (a) and TEM (b) of 1:8 Bi_2WO_6&Bi_2O_3 after 5 cycles of degradation experiments

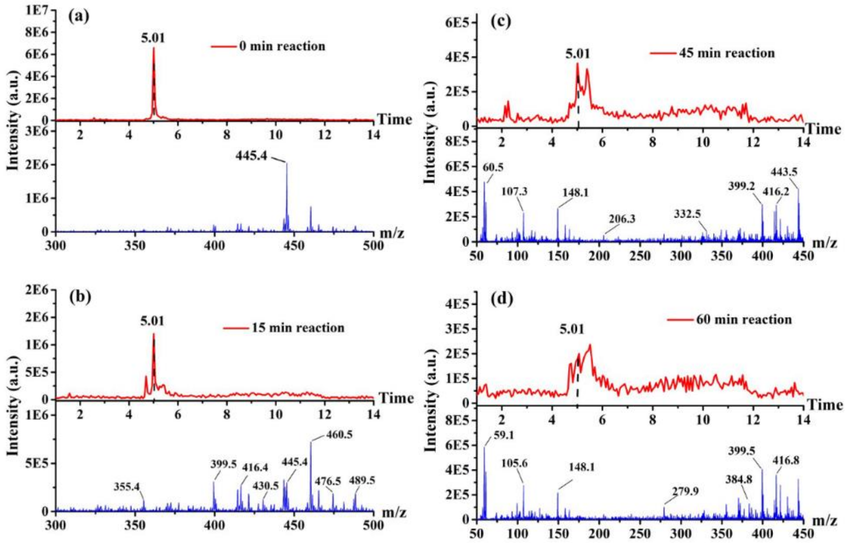

Figure S6 HPLC-MS/MS chromatograms of Tetracycline Hydrochloride degradation

products with 1:8 Bi_2WO_6&Bi_2O_3 catalyst：（a）0 min；（b）15 min；（c）45 min；（d）60 min

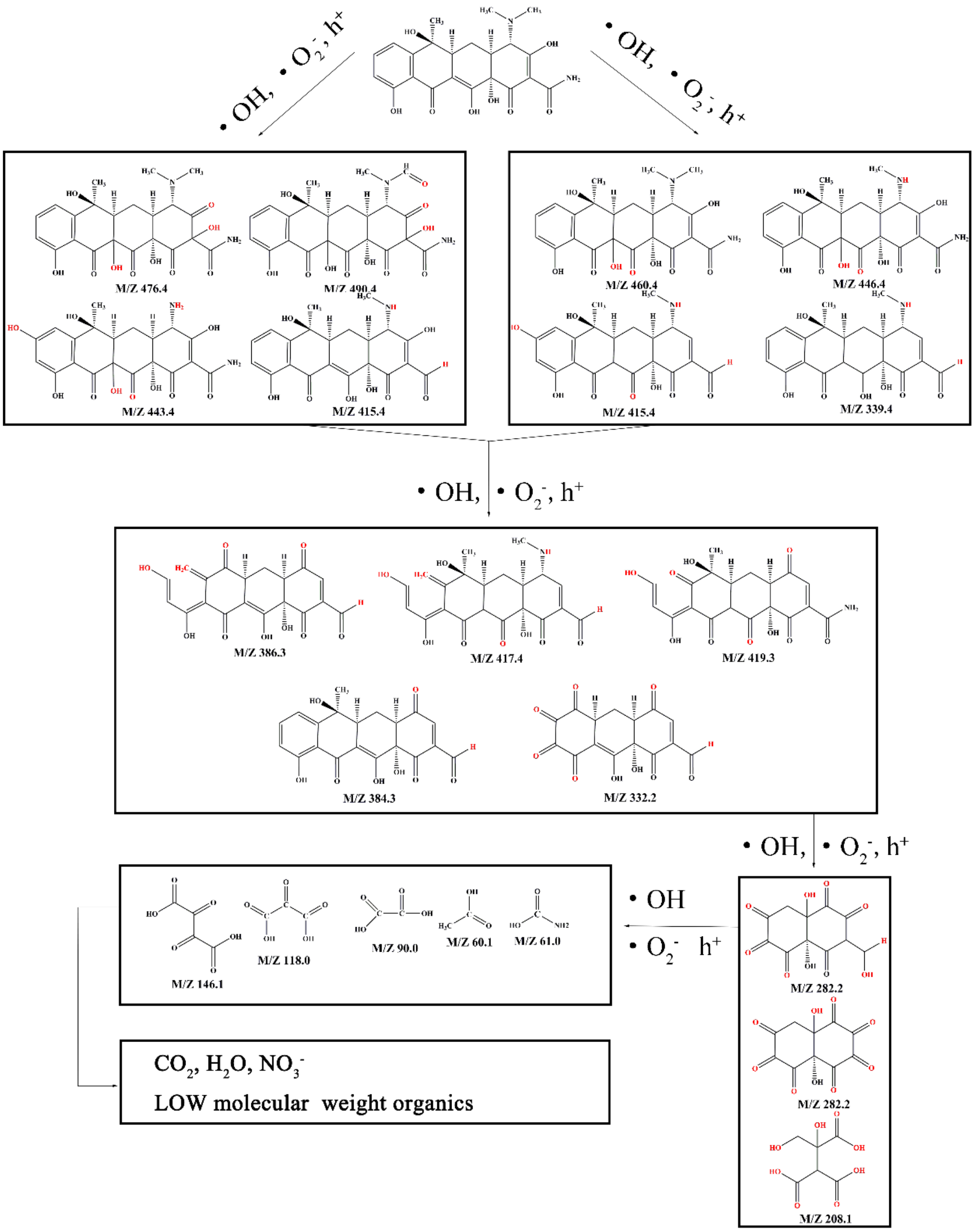

Figure S7 Proposed degradation pathway of TCH with the Bi_2WO_6&Bi_2O_3 catalyst

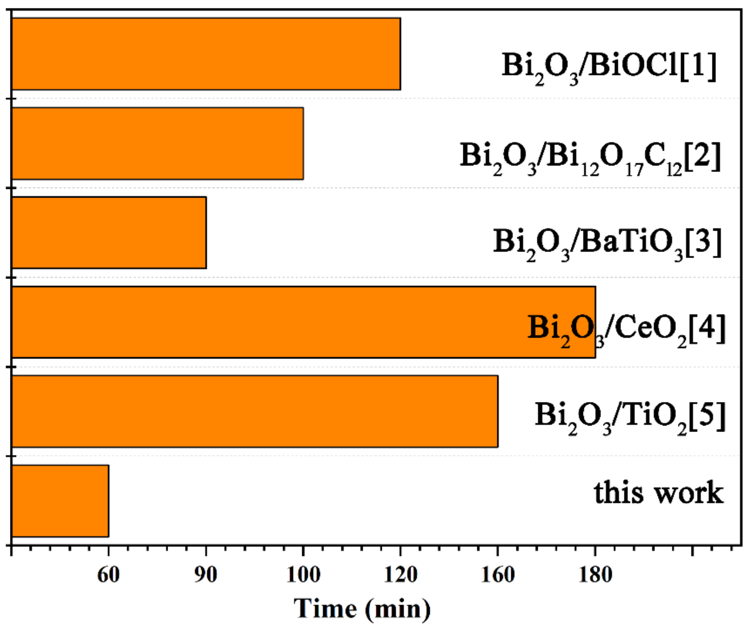

Figure S8 Degradation time of tetracycline by the 1:8 Bi_2WO_6&Bi_2O_3 of this work versus the same type of Bi_2O_3 composite catalyst in other works[27–31]（Catalyst : Tetracycline = 1mg : 60g）

Table-S2 Proposed degradation pathway of TCH with Bi2WO6/Bi2O3 catalyst

**Compound**	**Main Fragment （m/z）**	**Moleculr formula**	**Molecular structure**
TCH	444.4	$C_{22}H_{24}N_2O_8$	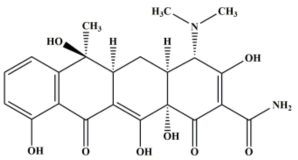
Product A	490.4	$C_{22}H_{22}N_2O_{11}$	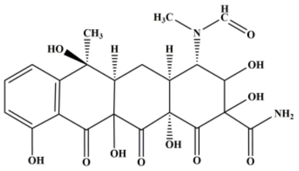

Product B	476.4	$C_{22}H_{24}N_2O_{10}$	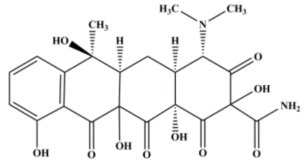
Product C	460.4	$C_{22}H_{24}N_2O_9$	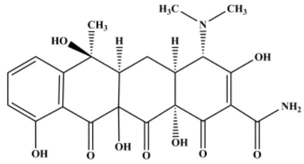
Product D	446.4	$C_{21}H_{22}N_2O_9$	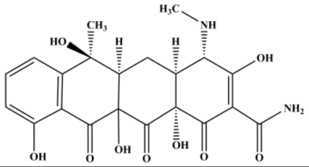
Product E	433.4	$C_{20}H_{19}N_2O_{10}$	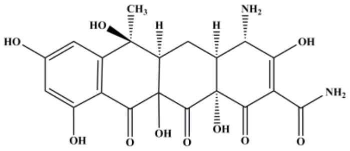
Product F	415.4	$C_{21}H_{21}N_1O_8$	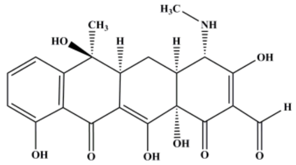
Product G	415.4	$C_{21}H_{21}N_1O_8$	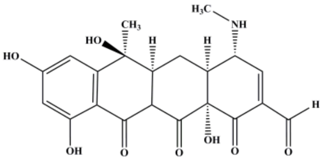
Product H	417.4	$C_{21}H_{23}N_1O_8$	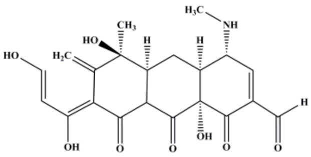
Product I	419.3	$C_{19}H_{17}N_1O_{10}$	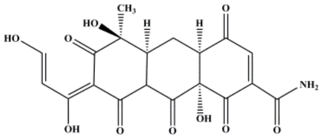
**Compound**	**Main Fragment （m/z）**	**Moleculr formula**	**Molecular structure**

Product J	399.4	$C_{21}H_{21}N_1O_7$	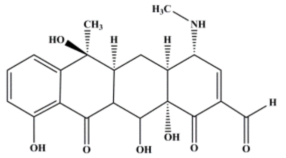
Product K	386.3	$C_{19}H_{14}O_9$	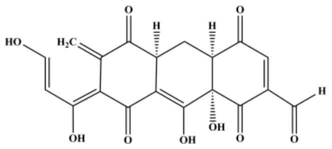
Product L	384.3	$C_{20}H_{16}O_8$	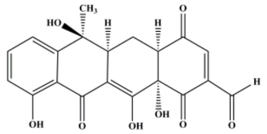
Product M	332.2	$C_{19}H_8O_9$	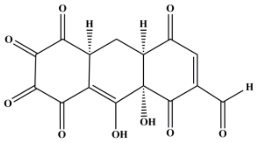
Product N	282.2	$C_{11}H_6O_9$	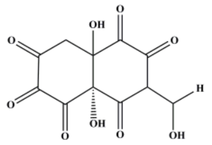
Product O	282.2	$C_{10}H_4O_9$	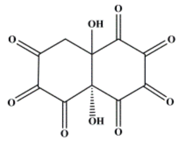
Product P	208.1	$C_6H_8O_8$	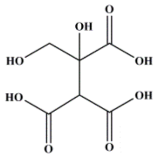
Product Q	146.1	$C_4H_2O_6$	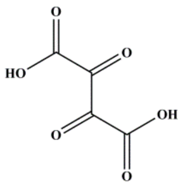
Product R	118.0	$C_3H_2O_5$	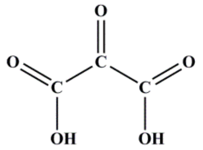
Product S	90.0	$C_2H_2O_4$	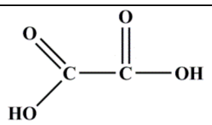

**Compoun**	**Main**	**Moleculr formula**	**Molecular structure**

d	Fragme nt （m/z）		
Product T	60.1	$C_2H_4O_2$	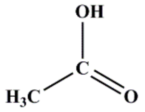
Product U	61.0	$C_3H_3NO_2$	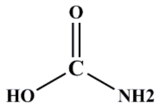

Table-S3 Changes in the pH of the solution during the degradation process of 1:8 Bi2WO6/Bi2O3 catalyst

pH	6.00	5.83	4.62	4.11	4.12	4.19
**Time(min)**	**0**	**10**	**20**	**30**	**40**	**50**

23. Kong S, An Z, Zhang W, An Z, Yuan M and Chen D (2019) Preparation of hollow flower-like microspherical β-bi2o3/BiOCL heterojunction and high photocatalytic property for tetracycline hydrochloride degradation. Nanomaterials (Basel, Switzerland) 10:57. doi: 10.3390/nano10010057

24. Chen J, Zhong J, Li J and Qiu K (2021) Boosted photocatalytic removal of tetracycline on S-scheme Bi12O17Cl2/α-Bi2O3 heterojunctions with rich oxygen vacancies. Applied surface science 563:150246. doi: 10.1016/j.apsusc.2021.150246

25. Zheng S, Ding B, Qian X, Yang Y, Mao L, Zheng S and Zhang J (2022) High efficiency degradation of tetracycline and rhodamine B using Z-type BaTiO3/γ-Bi2O3 heterojunction. Separation and purification technology 278:119666. doi: 10.1016/j.seppur.2021.119666

26. Yang X, Zhang Y, Wang Y, Xin C, Zhang P, Liu D, Mamba BB, Kefeni KK, Kuvarega AT and Gui J (2020) Hollow β-Bi2O3@CeO2 heterostructure microsphere with controllable crystal phase for efficient photocatalysis. Chemical engineering journal (Lausanne, Switzerland : 1996) 387:124100. doi: 10.1016/j.cej.2020.124100

27. Chen J, Tang T, Feng W, Liu X, Yin Z, Zhang X, Chen J and Cao S (2022) Large-Scale Synthesis of p–n Heterojunction Bi2O3/TiO2 Nanostructures as Photocatalysts for Removal of Antibiotics under Visible Light. ACS applied nano materials 5:1296-1307. doi: 10.1021/acsanm.1c03851
